# High-Pressure-Sprayed Double Stranded RNA Does Not Induce RNA Interference of a Reporter Gene

**DOI:** 10.3389/fpls.2020.534391

**Published:** 2020-12-16

**Authors:** Veli Vural Uslu, Alexandra Bassler, Gabi Krczal, Michael Wassenegger

**Affiliations:** ^1^AlPlanta-Institute for Plant Research, RLP AgroScience GmbH, Neustadt an der Weinstraße, Germany; ^2^Centre for Organismal Studies Heidelberg, Heidelberg University, Heidelberg, Germany

**Keywords:** double stranded RNA, small RNA sequencing, GFP silencing, RNA interference, RNA delivery

## Abstract

In plants, RNA interference (RNAi) is an effective defense mechanism against pathogens and pests. RNAi mainly involves the micro RNA and the small interfering RNA (siRNA) pathways. The latter pathway is generally based on the processing of long double stranded RNAs (dsRNA) into siRNAs by DICER-LIKE endonucleases (DCLs). SiRNAs are loaded onto ARGONAUTE proteins to constitute the RNA-induced silencing complex (RISC). Natural dsRNAs derive from transcription of inverted repeats or of specific RNA molecules that are transcribed by RNA-directed RNA polymerase 6 (RDR6). Moreover, replication of infecting viruses/viroids results in the production of dsRNA intermediates that can serve as substrates for DCLs. The high effectiveness of RNAi both locally and systemically implicated that plants could become resistant to pathogens, including viruses, through artificial activation of RNAi by topical exogenous application of dsRNA. The most preferable procedure to exploit RNAi would be to simply spray naked dsRNAs onto mature plants that are specific for the attacking pathogens serving as a substitute for pesticides applications. However, the plant cell wall is a difficult barrier to overcome and only few reports claim that topical application of naked dsRNA triggers RNAi in plants. Using a transgenic *Nicotiana benthamiana* line, we found that high-pressure-sprayed naked dsRNA did not induce silencing of a green fluorescence protein (GFP) reporter gene. Small RNA sequencing (sRNA-seq) of the samples from dsRNA sprayed leaves revealed that the dsRNA was, if at all, not efficiently processed into siRNAs indicating that the dsRNA was insufficiently taken up by plant cells.

## Introduction

Plant pests cause a significant decline in quantity and quality of crops as well as forestry products. The climate change alters the spreading of insect species, which may induce damage by feeding and/or by transmitting plant pathogens. For example, several studies have reported the recent invasion of Mediterranean plant pests like *Thaumetopoea processionea* in Northern European countries, including England, Denmark, and Sweden (Wagenhoff et al., 2014). Moreover, there are many regulatory restrictions on the use of conventional pesticides because of potential ecological and environmental hazard upon application (Robin and Marchand, 2019). Therefore and in view of the controversial discussions on the employment of genetically modified (gm) plants, novel versatile and gm-free eco-friendly approaches have become fundamental for pest control, including defense against viruses, in agriculture.

One of the strategies that plants, as sessile organisms, use to cope with pests is RNA interference (RNAi). RNAi comprises two main pathways: the micro RNA (miRNA) and the siRNA pathways (Ruiz-Ferrer and Voinnet, 2009; Borges and Martienssen, 2015). MiRNA production is initiated by transcription of endogenous miRNA genes, which are subsequently processed to typically 21-nt long miRNAs. Mature miRNAs bind to complementary transcripts for degradation or translational inhibition (Brodersen et al., 2008; Lanet et al., 2009; Li et al., 2018). It has been shown that plant-derived miRNAs, e.g., members of the miRNA 166 family, are taken up by aphids and this uptake correlates with resistance of melons to the aphid *Aphis gossypii* (Sattar et al., 2012).

The siRNA pathway is initiated by cleavage of double stranded RNA (dsRNA). DsRNA is subsequently processed into 21-nt, 22-nt, and 24-nt small interfering RNAs (siRNAs) (Fusaro et al., 2006). 21-nt and 22-nt siRNAs lead to post-transcriptional gene silencing mainly *via* degrading complementary transcripts, whereas 24-nt siRNAs mediate epigenetic modifications at complementary DNA for inducing transcriptional gene silencing (TGS) (Wassenegger et al., 1994; Wierzbicki et al., 2012; Dalakouras et al., 2020). The siRNA pathway blocks viral infections as well as transposable element activity. In addition, transgene expression is also frequently suppressed by siRNA-mediated TGS (Baulcombe, 2004).

Virus infections trigger RNAi upon formation of viral dsRNA replication intermediates or viral RNA secondary double stranded structures. Viral dsRNA is recognized by the RNA silencing machinery and is accordingly processed by Dicer-like enzymes (DCLs) into siRNAs. Argonaute (AGO) proteins binding these siRNAs to constitute the RNA-induced silencing complex (RISC) lead to the degradation of viral RNAs. Recruitment of RNA-directed RNA polymerase 6 (RDR6) to specific target RNAs (e.g., aberrant RNAs lacking a 5′ cap and/or a polyA-tail) leads to further dsRNA production (Dalmay et al., 2000; Vaistij et al., 2002; Gazzani et al., 2004). These dsRNAs are subsequently cleaved into secondary siRNAs, a process that is termed “transitivity.” Secondary siRNAs yield augmented defense against viruses and serve as footprints of the RNAi machinery (Baulcombe, 2004; Dunoyer and Voinnet, 2005). However, most viruses encode RNA silencing suppressors that impair the RNAi machinery by, for example, sequestering siRNAs or inhibiting AGOs (Silhavy and Burgyán, 2004). Hence, viral infection cannot be prevented by the plant defense in all cases.

In order to prevent virus infections it is essential to deliver dsRNA, exhibiting complementarity to the infecting virus already before the virus enters the plant cell. This strategy was successfully and numerously put into practice by the generation of gm plants expressing virus-specific RNAi-inducing transgene constructs (Wang et al., 2012; Pooggin, 2017). In recent years, alternative approaches that are based on exogenous delivery of dsRNA were employed to protect plants against virus infections. Exogenous RNAs of different origins such as *in vitro* and chemical synthesis or bacterial expression have been used (Lau et al., 2014; Dubrovina and Kiselev, 2019). These RNAs are delivered to plants using various methods, including low-pressure spraying, spreading by brushes, infiltration, biolistic approaches, trunk injections, mechanical inoculation, and high-pressure spraying (Dubrovina et al., 2019; Dalakouras et al., 2020). These methods appeared to improve plant defense against viruses slightly (Carbonell et al., 2008; Gan et al., 2010; Yin et al., 2010; Konakalla et al., 2016; Kaldis et al., 2018).

Different classes of adjuvants, including cationic nanoparticles, surfactants, clay nanosheets, peptide-based agents, and carbon dots have been used to boast plant defense against pests by facilitating the delivery of exogenous dsRNAs through the cell wall and subsequently cell membrane (Jiang et al., 2014; Mitter et al., 2017a; Schwartz et al., 2019; Worrall et al., 2019; Zheng et al., 2019). Indeed, it has been shown that these adjuvants improved plant defense against virus infection by increasing the uptake of dsRNA into plant cells and by protecting the dsRNAs from early degradation (Unnamalai et al., 2004; Mitter et al., 2017b).

Beside improving plant defense, it has been shown that naked dsRNA can be taken up by plant cells reducing the expression of transgenes in *Arabidopsis thaliana* (Mitter et al., 2017b; Dubrovina et al., 2019). However, the lack of molecular fingerprints of RNAi such as phased siRNAs in target sequences upon exogenous dsRNA applications raises questions about the underlying activity mechanism of the exogenous dsRNAs (Uslu and Wassenegger, 2020).

In this study, we investigated the RNA silencing efficacy of exogenously applied dsRNA and the processing of the dsRNA into siRNAs by the plant RNAi machinery using deep sequencing. For this purpose, the green fluorescence protein (GFP) -expressing *Nicotiana benthamiana* line 16C (Nb-16C) as a highly sensitive RNAi reporter system was treated with exogenous dsRNA to search for processed dsRNAs. *N. benthamiana* wild type (Nb-WT) plants were taken as controls to filter out the degradation products of the sprayed dsRNAs and water sprayed Nb-16C plants to eliminate the degradation products of endogenous target sequence. In order to deliver the dsRNAs into the plant cells, we employed the high-pressure spraying procedure (HPSP), which is reported to be the only method inducing transgene silencing via efficient activation of RNAi in *N. benthamiana* (Dalakouras et al., 2016, 2018). In this study, we demonstrate that dsRNA delivery by HPSP did not induce transgene silencing. In concordance with these finding, sRNA-seq revealed that the dsRNAs were not processed into specific siRNAs by RNAi machinery.

## Results

### DsRNA Synthesis and Monitoring of GFP Expression Upon HPSP

322nt-long dsRNA (dsRNA-5′GFP) and 139nt-long dsRNA (dsRNA-midGFP) matching the GFP sequence (position 1 to 322 and 294 to 432, respectively) expressed in the transgenic *N. benthamiana* line 16C (Nb-16C) were synthesized *in vitro* and annealed subsequently. Single stranded (ss) RNA and possible DNA contaminations were eliminated by DNAse and RNase treatment (Supplementary Figure 1). As a positive control, 22nt-long synthetic siRNA#164 matching the GFP sequence (position 164 to 187) has been used (Dalakouras et al., 2016).

High-pressure spraying creates a radial gradient of pressure. The center of sprayed area has the highest pressure and leads to wounded areas with 2–3 mm radii. The further the distance from the central region, the lower the pressure gets. Therefore, the periphery of the sprayed area recapitulates foliar spraying whereas the center of the area is subjected to high-pressure spraying. Noteworthy that the integrity of the dsRNAs sprayed onto the walls of a 15 ml-falcon tube under six-bar pressure was not affected (Supplementary Figure 1).

Leaves and buds of Nb-16C plants have been sprayed with 200 μl of dsRNA-midGFP at four different concentrations (10, 20, 200, and 240 ng/μl). In addition, 200 μl of dsRNA-5′GFP has also been sprayed on Nb-16C plants leaves and buds at three different concentrations (24, 48, and 240 ng/μl). As a positive control 200 μl of 22nt long synthetic siRNA#164 was sprayed at two different concentrations (1.4 and 14 ng/μl) onto the leaves and buds of Nb-16C of the same stage. 200 μl of water spraying is used as a negative control for GFP silencing.

**FIGURE 1:**
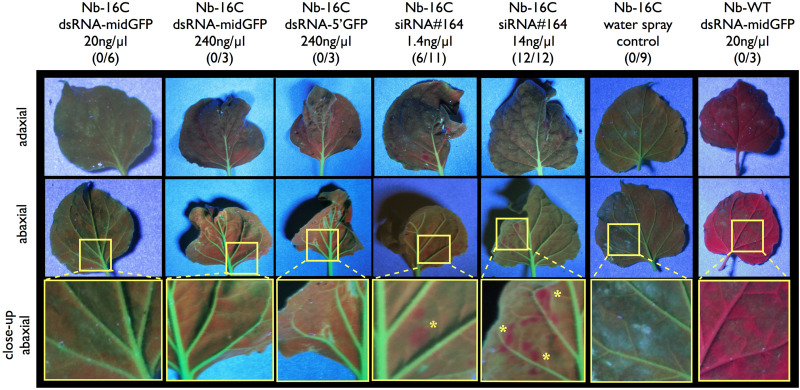
UV-light monitoring of GFP expression after dsRNA and siRNA spraying. Nb-16C plants were sprayed with 20 ng/μl dsRNA-midGFP, 240 ng/μl dsRNA-midGFP, 240 ng/μl dsRNA-GFP5′, 1.4 ng/μl siRNA#164, 14 ng/μl siRNA#164, water only and Nb-WT plants were sprayed with 20 ng/μl dsRNA-midGFP. One to four leaves per plant were sprayed. The three rows show adaxial and abaxial sides of matching leaves, and close up views of abaxial sides, visualized under the UV light, respectively. The area shown in close-up view is shown in yellow rectangle in abaxial view. Nb-WT sample is completely red due to the chloroplasts and Nb-16C samples are green due to the presence of GFP. Only the positive control leaves sprayed with low and high concentration of siRNA#164 showed silencing spots, highlighted with yellow stars in the close-up view. The number of plants showing silencing over the total number of plants treated in given condition is given in parenthesis.

Green Fluorescence Protein expression in sprayed plants was monitored under UV-light for in total 3 weeks. Since the early silencing in the positive controls takes place in a very restricted area, which is less than 5% of the leaf surface and the tissue damage is variable across different leaves, silencing phenotype was evaluated only qualitatively based on silenced spots but not quantitatively (Figure 1). Starting from 3 days and more visibly 5 days after spraying 6/11 of the positive controls with 1.4 ng/μl (0.1 μM) siRNA#164 and 12/12 of the positive controls with 14 ng/μl (1 μM) siRNA#164 showed local silencing spots (Figure 1). On the other hand, none of the samples sprayed with dsRNA-midGFP (0/15), dsRNA-5′GFP (0/9) or water (0/9) showed silencing up to 3 weeks after spraying (Figure 1). In order to understand whether the processed dsRNAs could not induce silencing or the dsRNAs were not processed to siRNAs in the first place, we performed an sRNA-seq experiment.

### Small RNA Sequencing

Leaf material from Nb-16C sprayed with water only and with 200 μl of 20 ng/μl dsRNA-midGFP has been collected 5 days post spraying (dps) for small RNA sequencing (sRNA-seq). As control leaf materials, three wildtype (WT) *N. benthamiana* plants sprayed with 200 μl of 20 ng/μl dsRNA-midGFP has been used (WT-ds). Due to the absence of the GFP transgene, WT plants, in contrast to Nb-16C plants, lack the potential to produce RDR6-transcribed secondary dsRNA, which are cleaved into secondary siRNAs by DCLs. Thus, in sprayed WT plants, secondary siRNA cannot accumulate and all the small RNAs matching the GFP sequence must be degradation product of the sprayed dsRNA-midGFP outside the leaf cells. One Nb-16C plant was sprayed with water to see the degradation products endogenously expressed GFP.

**FIGURE 2:**
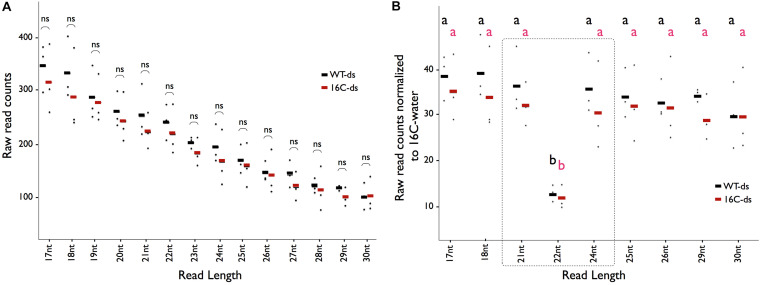
Distribution of sRNAs matching the GFP sequence. **(A)** The average abundance of sRNA-seq reads mapping to the GFP sequence for the given read length (*X*-axis) in WT-ds (black), 16C-ds (red) lines. Each dot represents one biological replicate in the given condition. Student *t*-test shows that there is no significant (ns) enrichment of an sRNA in 16C-ds samples when compared to WT-ds samples. **(B)** The number of reads with specific length mapping to the GFP in 16C-ds (red) and WT-ds (black) samples normalized to 16C-water samples does not show any enrichment of a specific class of sRNA. One-Way ANOVA test shows that there is only depletion of 22nt-long siRNA but no enrichment of sRNAs associated with gene silencing (shown in the dotted square). All data are based on the evaluation of three biological replicates and each dot represents one data point. Significant differences (a and b) were calculated by one-way ANOVA, Bonferroni’s post test *P* < 0.05.

Small RNA sequencing reads mapping to the GFP sequence in the dsRNA-midGFP-sprayed Nb-16C (16C-ds) and the dsRNA-midGFP-sprayed WT (WT-ds) samples exhibited an exponential decay curve resulting in higher read counts of shorter read lengths (*R* > 0.99 for all samples) (Supplementary Figure 2). Comparison of the reads of 16C-ds samples with same size reads of WT-ds did not show enrichment of any particular size of sRNAs, suggesting that no secondary siRNA was produced in 16C-ds plants (Figure 2A). When the reads of 16C-ds and WT-ds samples mapping to the GFP sequence were normalized to 16C-w samples, specific accumulation of 21-nt, 22-nt, or 24-nt RNAs was not detected, suggesting that neither in WT nor in Nb-16C, the sprayed dsRNA-midGFP was processed by DCLs (Figure 2B).

**FIGURE 3:**
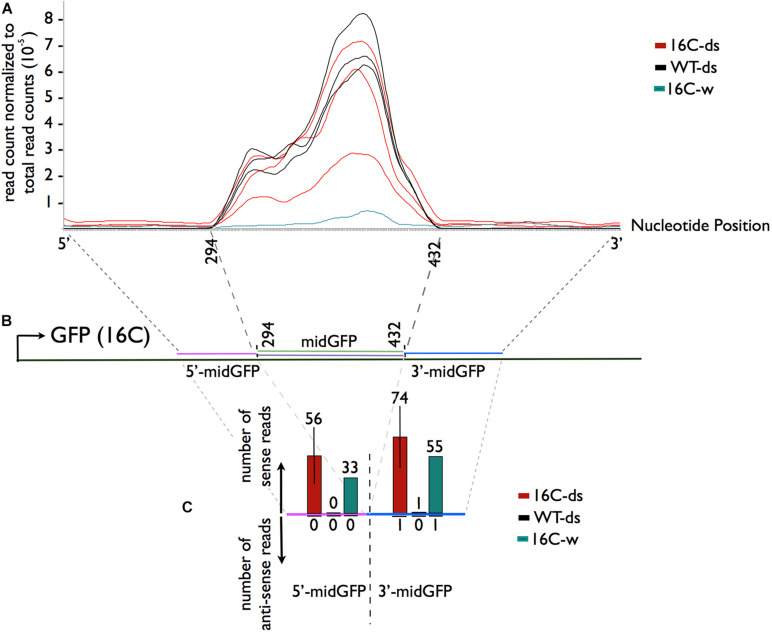
Sequence specific distribution of sRNAs. **(A,B)** SRNA reads mapping to the midGFP region and to the 5′ (magenta) and 3′ (blue) regions flanking the midGFP sequence regardless of the direction were normalized to the total read counts. There is no enrichment of 16C-ds reads over the WT-ds reads in the midGFP region, suggesting that there is no further amplification of the sRNAs in the presence of GFP transcript in 16C-ds. **(C)** The average raw read counts in the neighboring regions were shown to analyze transitivity. There are no anti-sense reads accumulating in 16c-ds samples outside of the sprayed area (downward arrow). The reads matching to the sense strand are degradation products of the GFP transcript, as they can be observed in 16-w sample but not WT-ds samples (upward arrow).

Comparison of normalized read count profiles between WT-ds and 16C-ds in midGFP region reads did not show any major differences, ruling out an amplification of siRNAs even in the presence of GFP target sequence in 16C-ds plants (Figures 3A,B). More importantly, a very characteristic indication of siRNA production is transitivity, which appears as phased siRNAs mapping to the adjacent sites of the target sequence on both strands. However, when sRNAs mapping to the 5′ and 3′ neighboring regions of midGFP were investigated in 16C-ds samples, there was clearly no accumulation of phased siRNAs outside of midGFP area, ruling out secondary siRNA-mediated transitivity (Figure 3C and Supplementary Figure 3). The sRNAs mapping to the GFP sequence outside of midGFP area were simply degradations products of endogenous GFP as they were observed in water sprayed 16C (16C-w) samples (Figure 3C).

## Discussion

In the face of climate change, current public opinion on the commercialization of transgenic plants, and regulatory restrictions on conventional pesticides, exogenous dsRNA-based applications gain further importance for pest control including viruses. However, the presence of an intact cell wall makes the delivery of dsRNAs into the plant cells challenging. In recent studies it has been claimed that naked dsRNAs can be taken up by intact leaf cells by foliar spraying or by simply spreading it by a brush. The dsRNAs delivered to plants subsequently led to transgene silencing and viral resistance. However, due to the fact that no siRNAs could be detected by sRNA-seq upon dsRNA treatment, the precise nature of the mode of action of exogenous dsRNA remains elusive (Mitter et al., 2017a). Therefore, in this study, we addressed the effectiveness of dsRNA delivery into mature plant leaves and analyzed if the dsRNA is processed into siRNAs by combining sRNA-seq and our well-established high-pressure spraying protocol in *N. benthamiana*.

For qualitative silencing analysis by visualizing GFP expressing plants under UV light, we used up to 26-times higher concentration of 139-nt dsRNA and 11 times higher concentration of 322nt dsRNA when compared to the 22-nt siRNA, which was sprayed as a low-concentration positive control (1.4 ng/μl). Considering that one molecule of 139nt dsRNA can be processed into 6 phased molecules of 22-nt siRNAs and 322nt dsRNA into 14 phased sRNA molecules of 22-nt siRNA, the effective dsRNA molarities were roughly 150 times more than the low concentration positive control siRNA. Yet, only the spraying of 22-nt siRNAs led to GFP transgene silencing in Nb-16C (Figure 1).

For sRNA-seq, 200 μl of 20 ng/μl (0.22 μM) dsRNA-midGFP was used for spraying three 16C and three WT *N*. *benthamiana*. At the initial step of RNAi, DCLs process dsRNAs into 21-, 22-, or 24-nt long distinct siRNAs. However, sRNA-seq showed the distribution of these particular sRNAs in 16C-ds and WT-ds was almost uniform when compared to the 16C-w with the exception of significantly lower 22-nt long siRNAs (Figure 2B). Since the whole leaf was used for sRNA extraction, this suggests that the initial step of RNAi upon dsRNA-midGFP spraying took place neither in the high-pressure (central) nor in the low-pressure sprayed areas (peripheral).

The RNAi machinery has the potential to amplify the plant defense by producing secondary siRNAs through RDR6 activity in the presence of a target complementary sequence, which in this case, is the GFP. Comparing WT-ds and 16C-ds samples allowed us to separate the contribution of the primary siRNAs, which are direct cleavage products of the sprayed dsRNA-midGFP, and secondary siRNAs, which are derive from cleavage products of the RDR6 transcribed secondary dsRNA-midGFP. However, this comparison showed that the presence of GFP target in 16C-ds sample did not increase the production of secondary siRNAs. Moreover, secondary siRNAs display transitivity and thus they map to the regions outside of the trigger dsRNA region. Previously, transitive secondary siRNAs were detected 6 days post agrobacterium mediated infiltration on 16C plants (Dalakouras et al., 2019). Therefore, we focused on early establishment of transitivity at 5 dps in this work. However, we couldn’t detect any sRNAs mapping to the complementary strand outside of the dsRNA-midGFP area (Figure 3C and Supplementary Figure 3). SRNAs mapping to the leading strand of the GFP in 16C-ds samples were detectable. However, these sRNAs derived from degradation of the GFP mRNA, since they were also present in the 16C-w sample.

In addition, the ratio of the longer reads (>24 nt) to shorter reads (<25 nt) was significantly higher outside of the dsRNA-midGFP area when compared to the dsRNA-midGFP area (Supplementary Figure 4). This observation suggests that the degradation of the exogenously delivered dsRNA differs significantly from degradation of the endogenous GFP mRNA.

Previous studies showed transgene silencing *via* dsRNA application in *A. thaliana* but we have not observed this phenomenon in *N. benthamiana* (Mitter et al., 2017b; Dubrovina et al., 2019). One possible explanation is that differences in the anatomy of the leaves and structure of the cell wall between *A. thaliana* and *N. benthamiana* led to contradictory results. However, in the same line with our results, the absence of siRNAs after bioclay-associated dsRNAs delivery in *Nicotiana tabacum* suggests that dsRNA-based transgene silencing, as well as plant protection against pests and viruses may be an indirect effect of the dsRNA.

Despite being a promising approach for plant protection, the mechanisms underlying the effect of exogenous dsRNA application on viral resistance, pest control, and transgene silencing remain controversial and elusive. However, successful applications of new generation adjuvants, e.g., carbon dots are promising approaches for improving dsRNA delivery and efficient pest control in the near future.

## Materials and Methods

### Synthesis and Purification of dsRNA

The 139bp-long GFP-mid fragment was amplified using the GFP139-F (*TAATACGACTCACTATAGGGAGA*gtcgacTATGA AGCGGCACGACTTCT) and the GFP139-R (*TAATACGACT CACTATAGGGAGA*gagctcGATCCTGTTGACGAGGGTGT) primers, both containing a 23 bp long *T7 promoter* and a *Sal*I (5′ end, GFP139-F) and *Sac*I (3′ end, GFP139-R) recognition sequences. The 322bp-long GFP-5′ fragment as amplified using GFP5′-F (*TAATACGACTCACTATAGGGAGA* ATGAAGACTAATCTTTTTCTCTTT) and GFP5′-R (TAATA CGACTCACTATAGGGAGACTCAGGCATGGCGCTCTTGA) primers, both containing the 23bp-long *T7 promoter.*

Both PCR products (200 ng) were used as a template to produce dsRNA using the MEGAscript^®^ RNAi Kit^1^ according to manufacturer’s instructions. DNA template and single stranded RNAs were digested with DNaseI and RNase (provided by the kit) for 1 h. dsRNA was purified using a filter cartridge (provided by the kit) and eluted in 10 mM Tris–HCl buffer containing 1 mM EDTA (pH = 7.0). In six reactions, 358.4 μg of dsRNA-midGFP was produced in total (33.4, 36.7, 36.1, 35.3, 99.1, and 117.8 μg). 227.0 μg of dsRNA-GFP-5′ was synthesized in three reactions combined (80.6, 30.7, and 115.7 μg).

### High-Pressure Spraying

For each plant, 200 μl of aqueous dsRNA solutions at given concentrations were sprayed from a 0.5–1 cm distance at the abaxial surface of leaves with an airbrush pistol (CONRAD AFC-250A, 0.25 mm nozzle)^2^ and at a pressure of 5–6 bar provided by the METABO Elektra Beckum Classic 250 compressor^3^. 10–12 cm tall *N. benthamiana* wildtype and Nb-16C plants were sprayed with dsRNA and as a control Nb-16C plants were sprayed with water, using the same airbrush type. Each treatment was conducted with a separate airbrush to avoid cross contamination. 11 Nb-16C plants were sprayed with 1.4 ng/μl siRNA#164, 12 plants were sprayed with 14 ng/μl siRNA#164, three plants with 10 ng/μl dsRNA-midGFP, six plants with 20 ng/μl dsRNA-midGFP, three plants with 200 ng/μl dsRNA-midGFP, three plants with 240 ng/μl dsRNA-midGFP, three plants with 24 ng/μl dsRNA-5′GFP, three plants with 48 ng/μl dsRNA-5′GFP, three plants with 240 ng/μl dsRNA-5′GFP, nine plants only with water for monitoring silencing under UV-light. For each plant, 1–4 leaves and one apical meristem bud were sprayed.

### RNA Extraction and Small RNA Sequencing

Two leaves per plant from three Nb-16C plants and three Nb-WT plants, sprayed with 20 ng/μl dsRNA-midGFP and two leaves from one Nb-16C sprayed with water were harvested for RNA 5 dps using the mirVana miRNA extraction kit (see text footnote 1) according to manufacturer’s instructions. For 16C-ds and WT-ds, three biological replicates were sequenced and evaluated for the experiments and one 16C-w sample was sequenced for normalization purposes (Figures 2B, 3C). 250 ng of RNA per sample was used in library preparation and small RNA libraries were prepared by GenXPro GmbH using the TrueQuant SmallRNA Seq Kit according to the manual of the manufacturers (GenXPro GmbH, Germany). The libraries were sequenced on an Illumina NextSeq500 instrument using 75 cycles of sequencing. sRNA-seq quality control was performed by plotting the read counts of sRNAs longer than 16 bp and shorter than 30 bps. The accumulation of 24-nt long and 21-nt long sRNAs in both Nb-16C and Nb-WT show that the sequencing quality is good and consistent among different samples (Supplementary Figure 5).

### Bioinformatic Analysis

Small RNA sequencing reads in FASTQ files are used to filter out the 3′ sequencing adapter and quantified FASTA files are obtained. FASTA reads are mapped to the 16C-GFP and midGFP sequences and number of reads per region of interest is measured (Philips et al., 2017). TABLET software was used for qualitative analysis of the data and graphical representation of the mapped sRNA reads (Milne et al., 2013; Supplementary Figure 3). Total read counts and mapped read counts are given in Table 1.

**TABLE 1:** Samples analyzed by sRNA-seq, the total read numbers and specific reads of specific sized mapping to the region of interest of GFP, indicated in Figure 2.

Sample name	Total number of reads	Reads matching to the ROI	20nt-long	21nt-long	22nt-long	23nt-long	24nt-long	25nt-long
16C-ds_1	7186316		208	194	186	162	152	156
16C-ds_2	7511064		299	260	276	214	229	204
16C-ds_3	7375715		230	222	202	179	127	122
WT-ds_1	8363721		300	314	275	214	239	201
WT-ds_2	8824820		237	234	246	209	171	164
WT-ds_3	8434532		249	220	209	193	182	148
16C-w	7718149		9	14	37	5	11	10

### SRNA-Seq Quantification and Normalization

The comparison of siRNAs mapping to the GFP was done based on raw read counts without any normalization (Figure 2A). The enrichment analysis of siRNAs of the given sizes was performed by dividing the raw read count numbers mapping in 16C-ds and WT-ds to 16C-w (Figure 2B). The normalized read count at a given position and sample is calculated by the average read count in a sliding window of ten nucleotides divided by the total read count of the given sample (Figure 3).

However, there are no established protocols for normalization for quantifying the efficiency of dsRNA processing into siRNAs upon HPSP. Considering that the amount of dsRNA on the leaf surface may alter by the fluctuations in the pressure, the angle, the distance, and the duration of spraying an optimal normalization approach reflecting the efficiency of dsRNA processing into siRNA is lacking. Therefore, additional normalization tests were performed (Supplementary Figure 6). For a functional normalization, we took the reads mapping to miR159 as a reference, because miR159 is also processed by RNAi machinery that is also involved in the cleavage of dsRNA (Supplementary Figure 6A). In addition, we used all 24-nt long-reads as a global functional normalization reference for each sample (Supplementary Figure 6B) as most of 24-nt reads are also products of RNAi machinery.

### Statistical Analysis

Pairwise comparisons between same length reads counts of 16C-ds and WT-ds was performed by student *t*-test with significance cut-off of *p* < 0.05 (Figure 2A). Statistical comparison among multiple normalized read counts of 16C-ds and WT-ds has been performed with One-Way ANOVA test with Bonferroni post-test *p* < 0.05 (Figure 2B). The comparison of degradation products of exogenous and endogenous RNAs has been done with Fisher’s Exact Test (Supplementary Figure 4).

### Ultraviolet (UV) Monitoring

Green Fluorescence Protein fluorescence of Nb-16C plants was monitored using the Black-Ray B-100 UV Lamp^4^. At least three plants per treatment were analyzed. The photos are taken by Canon EOS700D (18–55 mm), aperture priority mode (*A* = 10).

## Data Availability Statement

The data discussed in this publication have been deposited in NCBI’s Gene Expression Omnibus (Edgar et al., 2002) and are accessible through GEO Series accession number GSE160110 (https://www.ncbi.nlm.nih.gov/geo/query/acc.cgi?acc=GSE160110).

## Author Contributions

VVU, GK, and MW conceived the experiments and wrote the manuscript. VVU and AB conducted the experiments. All authors contributed to the article and approved the submitted version.

## Conflict of Interest

The authors declare that the research was conducted in the absence of any commercial or financial relationships that could be construed as a potential conflict of interest.

## Footnotes

1 www.thermofisher.com

2 www.conrad.de

3 www.metabo.com

4 www.fishersci.de

## Supplementary Material

Supplementary Figure 1 DsRNA integrity after high pressure spraying. DsRNA-midGFP shows no indication for degradation and dissociation of the sense and antisense stands after spraying with a six bar pressure [lane (3)]. High-pressure sprayed dsRNA-midGFP was melted at 95°C for 4 min and rapidly cooled-down to show the gel electrophoresis pattern of dissociated sense and antisense strands [lane (4)]. The banding patterns indicated that the high-pressure sprayed dsRNA-midGFP [lane (3)] is composed of dsRNA just like, not sprayed dsRNA-midGFP [lane (2)] rather than ssRNA [lane (4)]. Low-range RNA ladder is loaded to lane (1).

Supplementary Figure 2 Exogenous dsRNA decay. The exponential decay curve of the average sRNA-seq reads of WT-ds (black line) and 16C-ds (red line) mapping to the GFP sequence. Both graphs are exponential with very high *R* value, in consistent with the lack of sRNAs of specific sizes such as 21, 22, and 24nt. Raw read counts are used as in Figure 2.

Supplementary Figure 3 Strand Specific Distribution of sRNAs. SRNA reads mapping to the midGFP region and to the 5′ (magenta) and 3′ (blue) regions flanking the midGFP sequence are shown. The reads aligning to the sense strand are shown in green and reads mapping to the antisense strand are show in purple. All three sequence samples (biological replicates) from 16C-ds to WT-ds are plotted. Antisense strand reads only match to the midGFP sequence. The graphical representations are screenshots obtained by TABLET software (Milne et al., 2013).

Supplementary Figure 4 Size distribution of the sense sRNAs mapping to the GFP within and outside of the midGFP area in 16C-ds. sRNAs mapping to sense midGFP sequence are predominantly shorter than 25-nt. These sRNAs possibly originate from the dsRNA-midGFP degradation on the leaf surface. However, when the midGFP area is excluded, the rest of the sense sRNA reads derived from the degradation of the GFP mRNAs are enriched for reads longer than 24-nt (<24 nt). All data are based on the evaluation of three biological replicates normalized to the <24-nt counts. The statistical comparison is performed by Fishers exact Test (*p* < 0.05).

Supplementary Figure 5 Quality control of the sRNA-seq experiment. The percent of the reads (*Y*-axis) of the given read length (*X*-axis) are plotted. There is a clear enrichment of 24nt-long and 21nt-long sRNAs in both WT-ds and 16C-ds, ensuring the quality of the sRNA-seq for sRNA analysis.

Supplementary Figure 6 Alternative normalization scheme for sRNA-analysis. **(A)** the reads matching to the midGFP area and the neighboring sites are normalized with respect to the miRNA159 level. miRNAs are also processed by the RNAi machinery, this normalization scheme is used as a functional normalization. **(B)** the reads matching to the midGFP area and the neighboring sites are normalized with respect to the total 24nt-long sRNA abundance. 24nt-long sRNAs are mostly also processed by RNAi machinery, therefore, this normalization scheme is used an alternative global functional normalization.
