# Supplementary figures and images for: High-Pressure-Sprayed Double Stranded RNA Does Not Induce RNA Interference of a Reporter Gene
Source: Front Plant Sci. 2020 Dec 16;11:534391. doi: 10.3389/fpls.2020.534391 (PMC7773025; doi:10.3389/fpls.2020.534391)

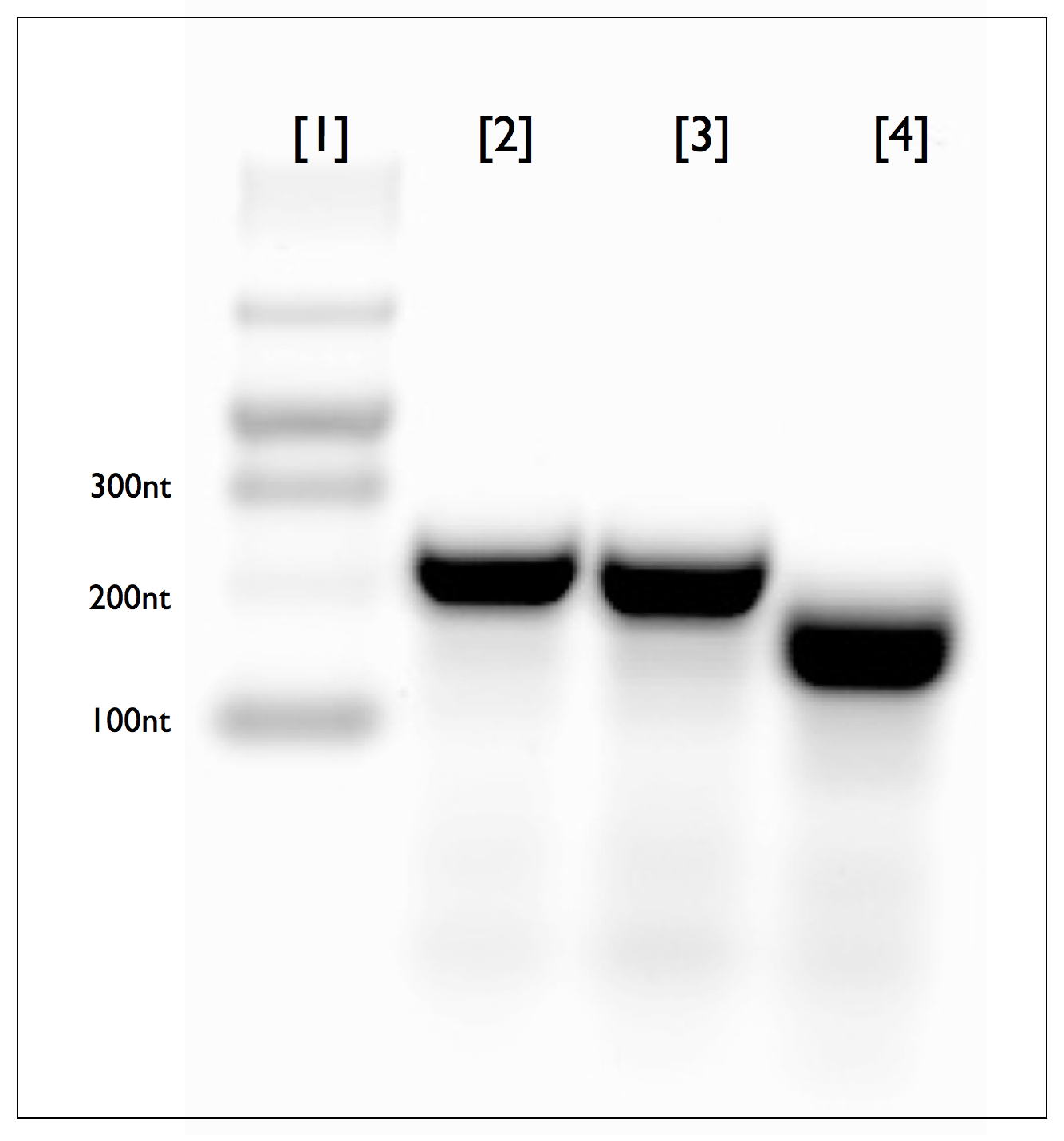

Supplement: Supplementary Figure 1 — DsRNA integrity after high pressure spraying. DsRNA-midGFP shows no indication for degradation and dissociation of the sense and antisense stands after spraying with a six bar pressure [lane (3)]. High-pressure sprayed dsRNA-midGFP was melted at 95°C for 4 min and rapidly cooled-down to show the gel electrophoresis pattern of dissociated sense and antisense strands [lane (4)]. The banding patterns indicated that the high-pressure sprayed dsRNA-midGFP [lane (3)] is composed of dsRNA just like, not sprayed dsRNA-midGFP [lane (2)] rather than ssRNA [lane (4)]. Low-range RNA ladder is loaded to lane (1). [file Image_1.JPEG]

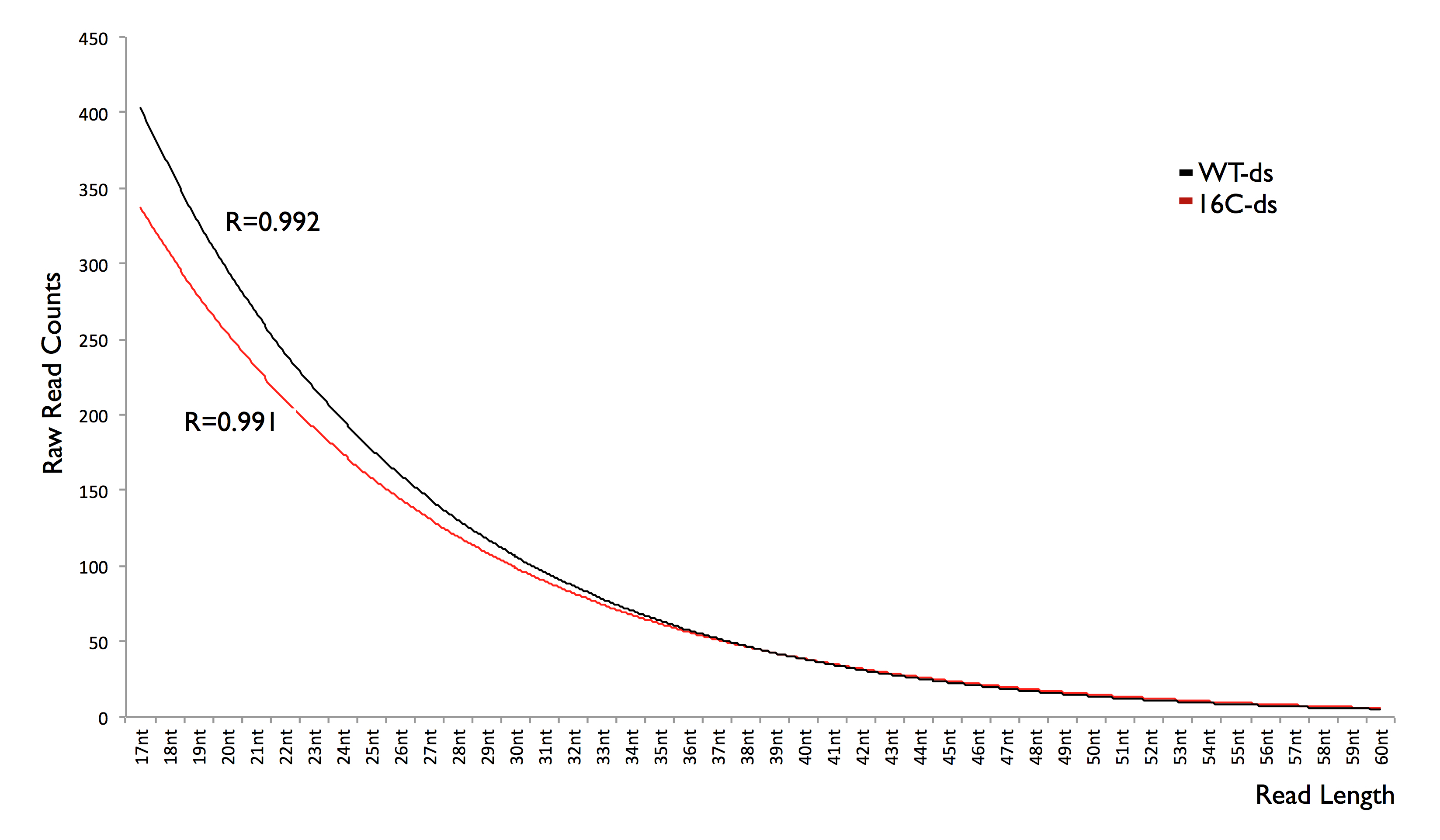

Supplement: Supplementary Figure 2 — Exogenous dsRNA decay. The exponential decay curve of the average sRNA-seq reads of WT-ds (black line) and 16C-ds (red line) mapping to the GFP sequence. Both graphs are exponential with very high R value, in consistent with the lack of sRNAs of specific sizes such as 21, 22, and 24nt. Raw read counts are used as in Figure 2. [file Image_2.JPEG]

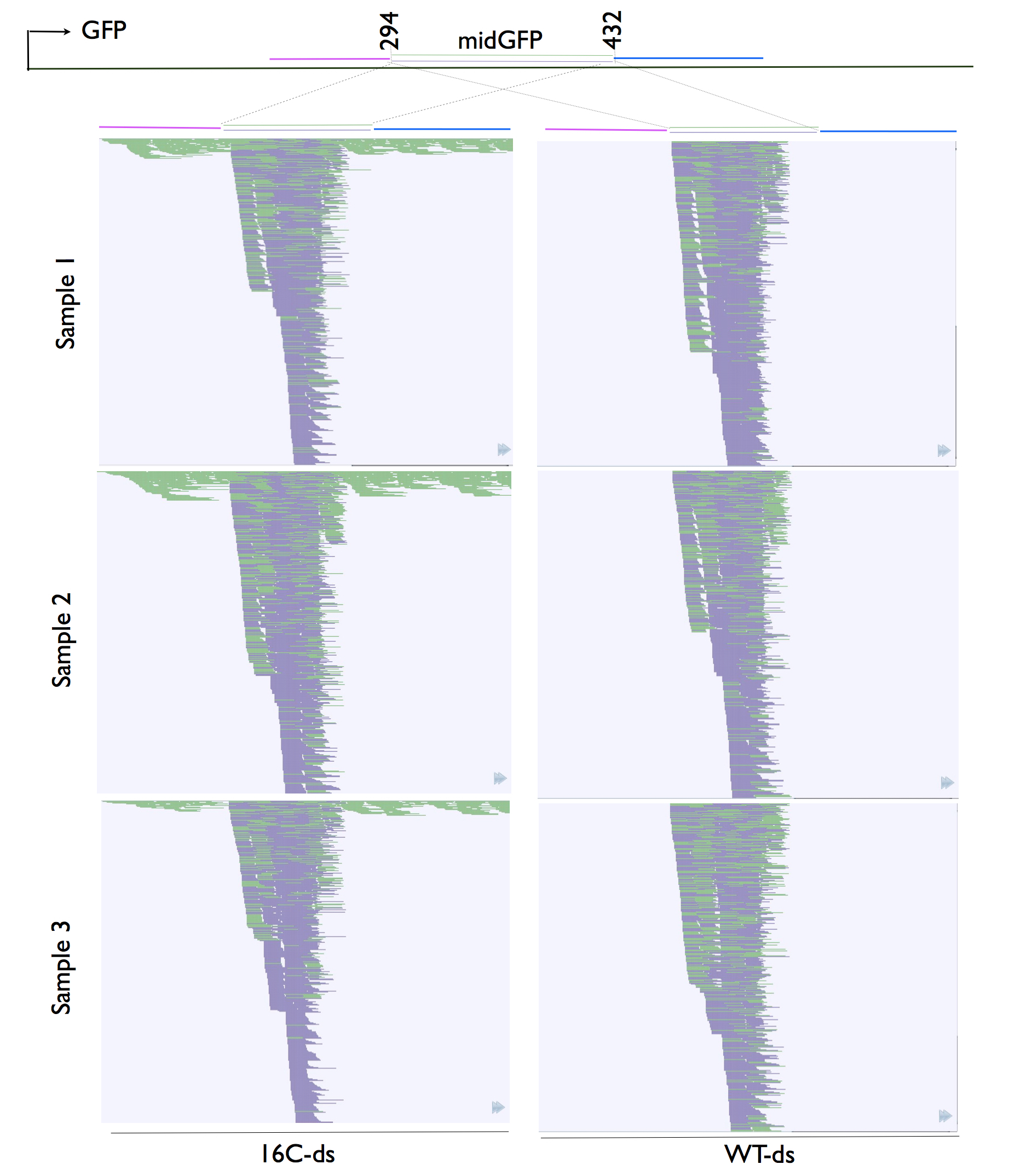

Supplement: Supplementary Figure 3 — Strand Specific Distribution of sRNAs. SRNA reads mapping to the midGFP region and to the 5′ (magenta) and 3′ (blue) regions flanking the midGFP sequence are shown. The reads aligning to the sense strand are shown in green and reads mapping to the antisense strand are show in purple. All three sequence samples (biological replicates) from 16C-ds to WT-ds are plotted. Antisense strand reads only match to the midGFP sequence. The graphical representations are screenshots obtained by TABLET software (Milne et al., 2013). [file Image_3.JPEG]

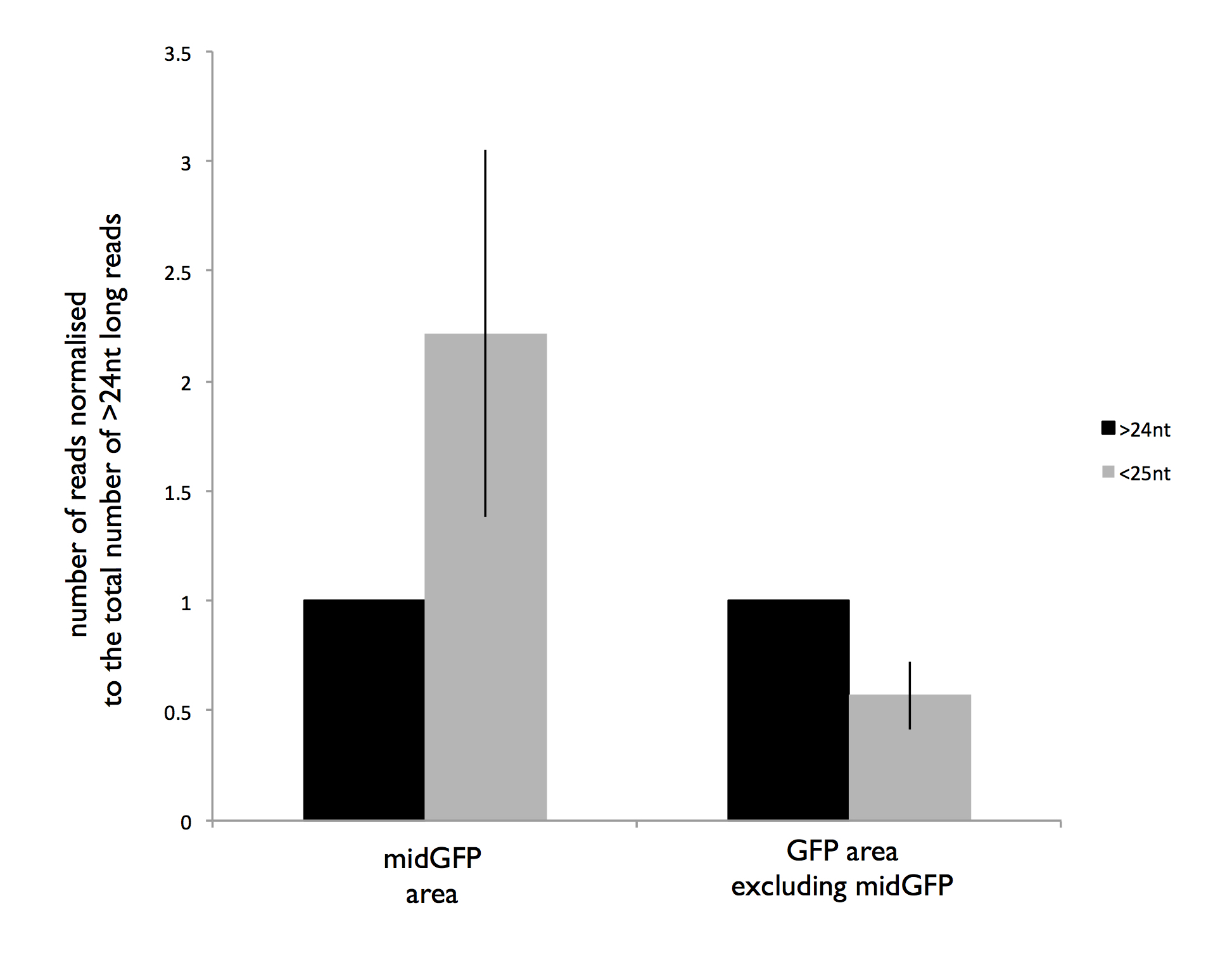

Supplement: Supplementary Figure 4 — Size distribution of the sense sRNAs mapping to the GFP within and outside of the midGFP area in 16C-ds. sRNAs mapping to sense midGFP sequence are predominantly shorter than 25-nt. These sRNAs possibly originate from the dsRNA-midGFP degradation on the leaf surface. However, when the midGFP area is excluded, the rest of the sense sRNA reads derived from the degradation of the GFP mRNAs are enriched for reads longer than 24-nt (<24 nt). All data are based on the evaluation of three biological replicates normalized to the <24-nt counts. The statistical comparison is performed by Fishers exact Test (p < 0.05). [file Image_4.JPEG]

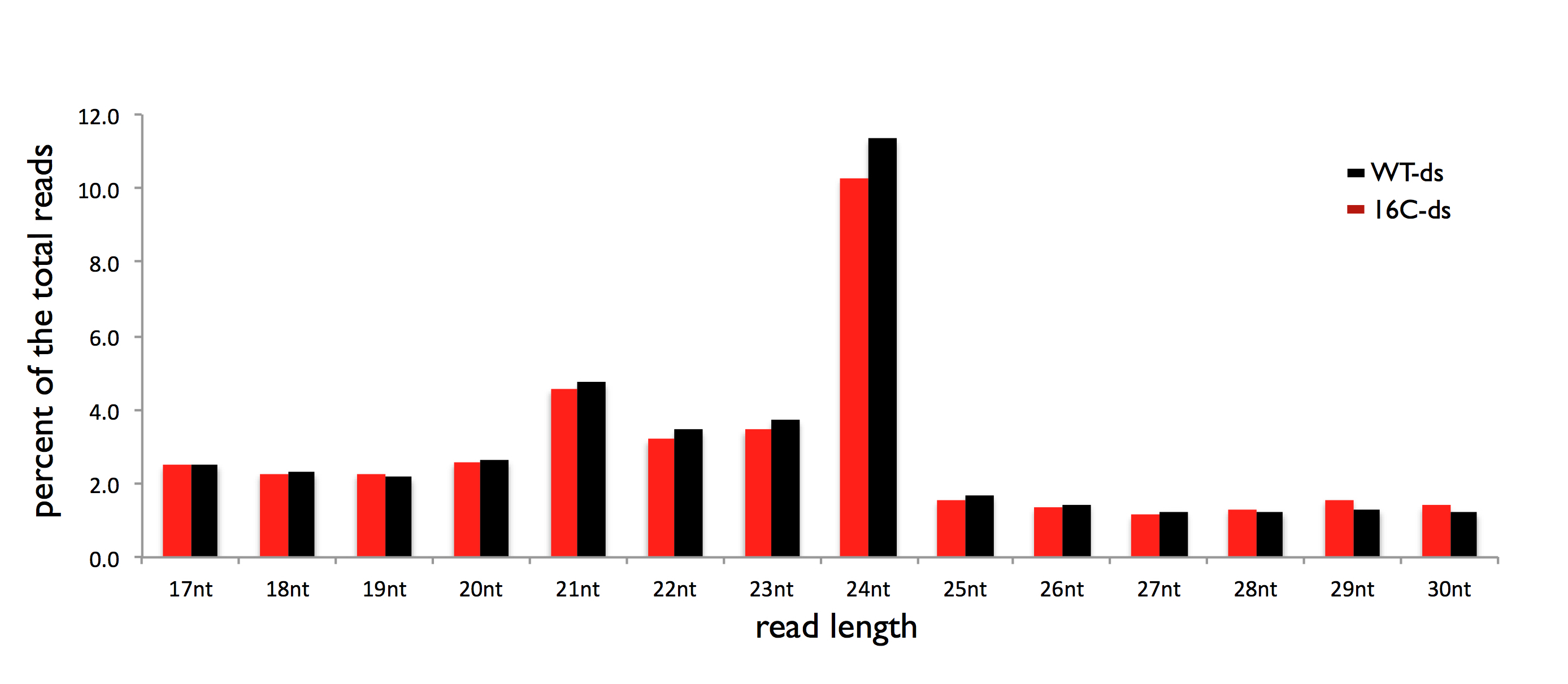

Supplement: Supplementary Figure 5 — Quality control of the sRNA-seq experiment. The percent of the reads (Y-axis) of the given read length (X-axis) are plotted. There is a clear enrichment of 24nt-long and 21nt-long sRNAs in both WT-ds and 16C-ds, ensuring the quality of the sRNA-seq for sRNA analysis. [file Image_5.JPEG]

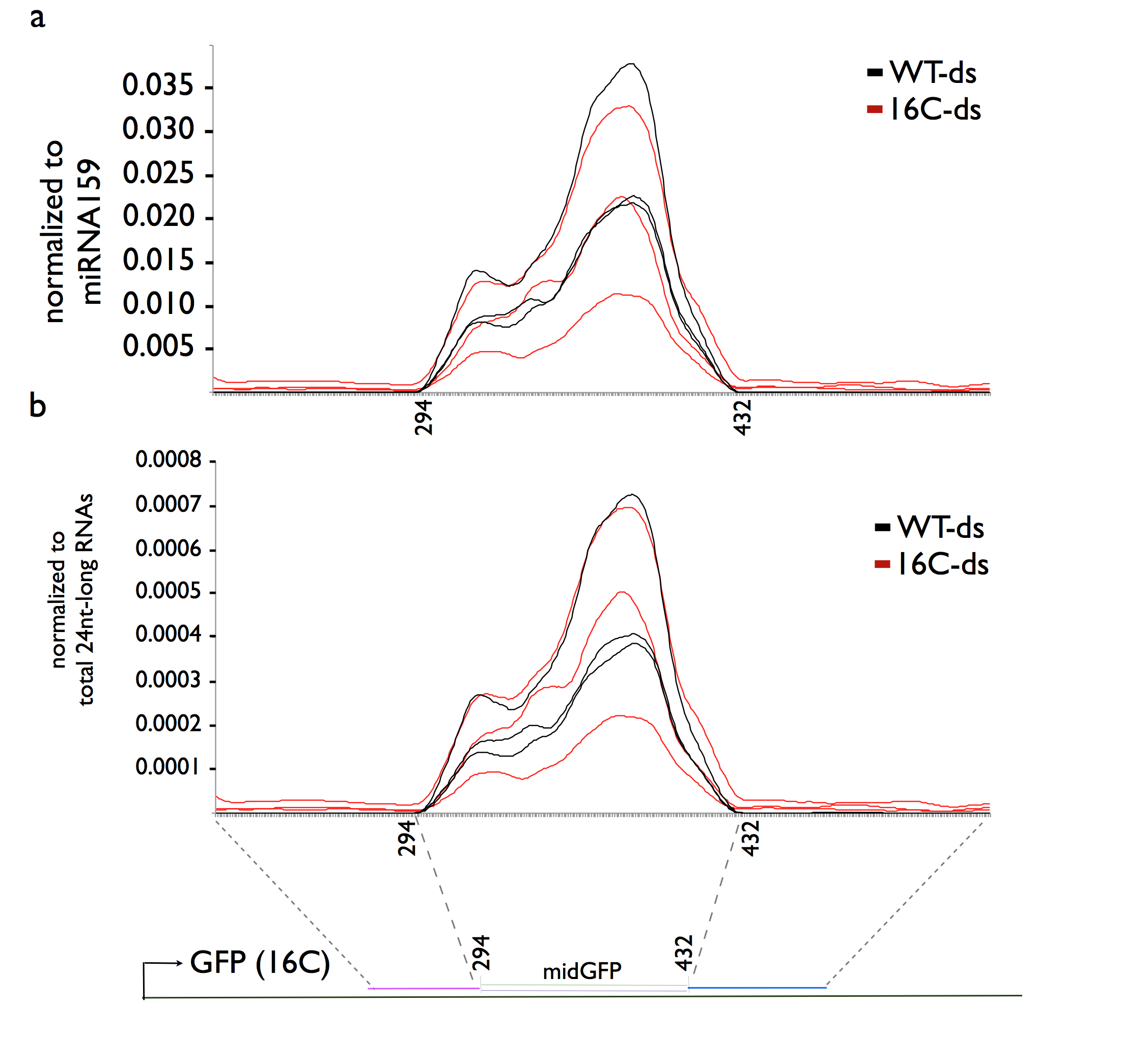

Supplement: Supplementary Figure 6 — Alternative normalization scheme for sRNA-analysis. (A) the reads matching to the midGFP area and the neighboring sites are normalized with respect to the miRNA159 level. miRNAs are also processed by the RNAi machinery, this normalization scheme is used as a functional normalization. (B) the reads matching to the midGFP area and the neighboring sites are normalized with respect to the total 24nt-long sRNA abundance. 24nt-long sRNAs are mostly also processed by RNAi machinery, therefore, this normalization scheme is used an alternative global functional normalization. [file Image_6.JPEG]
